# Correlation between Sialylation Status and Cell Susceptibility to Amyloid Toxicity

**DOI:** 10.3390/cells11040601

**Published:** 2022-02-09

**Authors:** Eleonora Sgambati, Alessia Tani, Manuela Leri, Giovanni Delfino, Sandra Zecchi-Orlandini, Monica Bucciantini, Daniele Nosi

**Affiliations:** 1 Department of Biosciences and Territory, University of Molise, Contrada Fonte Lappone, Pesche, 86090 Isernia, Italy; eleonora.sgambati@unimol.it; 2 Department of Experimental and Clinical Medicine, Section of Anatomy and Histology, University of Florence, Largo Brambilla 3, 50134 Florence, Italy; alessia.tani@unifi.it (A.T.); sandra.zecchi@unifi.it (S.Z.-O.); daniele.nosi@unifi.it (D.N.); 3 Department of Experimental and Clinical Biomedical Sciences “Mario Serio”, University of Florence, Viale Morgagni 50, 50134 Florence, Italy; manuela.leri@unifi.it; 4 Department of Biology (BIO), University of Florence, Via Giorgio La Pira 4, 50121 Florence, Italy; giovanni.delfino@unifi.it

**Keywords:** sialylation status, Aβ-42 toxicity, amyloid–plasma membrane interaction, cell differentiation, GM1

## Abstract

The interaction between the cell membrane and misfolded protein species plays a crucial role in the development of neurodegeneration. This study was designed to clarify the relationship between plasma membrane composition in terms of the differently linked sialic acid (Sia) content and cell susceptibility to toxic and misfolded Aβ-42 peptides. The sialylation status in different cell lines was investigated by lectin histochemistry and confocal immunofluorescence and then correlated with the different propensities to bind amyloid fibrils and with the relative cell susceptibility to amyloid damage. This study reveals that expressions of Sias α2,3 and α2,6 linked to galactose/N-acetyl-galactosamine, and PolySia are positively correlated with Aβ-42-induced cell toxicity. PolySia shows an early strong interaction with amyloid fibrils, favoring their binding to GM1 ganglioside containing α2,3 galactose-linked Sia and a loss of cell viability. Our findings demonstrate that cell lines with a prevailing plastic neuron-like phenotype and high monoSia and PolySia contents are highly susceptible to amyloid Aβ-42 toxicity. This toxicity may involve a change in neuron metabolism and promote a compensative/protective increase in PolySia, which, in turn, could favor amyloid binding to GM1, thus exacerbating cell dysmetabolism and further amyloid aggregation.

## 1. Introduction

Protein misfolding diseases (PMDs), including several neurodegenerative diseases and systemic amyloidosis, are caused by mutant misfolding-prone proteins that can neither be refolded nor cleared efficiently by the protein quality control (PQC) intracellular system. This deregulation leads them to form intracellular/extracellular accumulations into cytotoxic aggregates, characterized by the peculiar amyloid cross-β conformation. Indeed, PMDs share common structural and functional traits that contribute to pathogenetic events: (i) aggregate formation, (ii) PQC impairment, (iii) interaction of the aggregates with the membrane, (iv) cell-to-cell transmission of aggregates. Evidence highlights the general role of plasma membranes in recruiting proteins and peptide monomers and favoring their local concentration and aggregate nucleation [1,2,3]. Moreover, misfolded monomers, their prefibrillar aggregates and the aggregation process itself [4] may locally disarrange the packing order of the lipid bilayer and form non-specific passages or real ionic channels, thus affecting the asymmetric ion distribution and specific protein activity of the cell membrane. This is followed by impairment of intracellular Ca^2+^ homeostasis and oxidative stress. Therefore, interactions of misfolded oligomers and/or other unstable prefibrillar intermediates with the cell membrane are the first step of amyloid cytotoxicity [5]. Some studies reported that different types of misfolded oligomers of the same protein, grown under different destabilizing conditions, display their own physicochemical features, in terms of compactness and exposure of hydrophobic sites, which can be related to their different cytotoxicity degrees [6,7]. Moreover, amyloid toxicity is not inherent to a specific type of oligomer but represents an emerging property, arising from a complex interplay between conformations of amyloid intermediates and biophysical features of outer and inner cyto-membranes, which may vary in different cell types [3,7,8]. Distinctive deregulation of lipid homeostasis in plasma membranes has been demonstrated during physiological aging and neurodegenerative disorders, such as Parkinson’s disease (PD) and Alzheimer’s disease (AD) [9]. In particular, liquid-ordered phase microdomains in plasma membranes may undergo changes in lipid composition, consistent with neuronal impairment. These microdomains, rich in sphingolipids and cholesterol, are considered key points of signaling protein clusters. Lipid alterations in such signaling platforms may have consequences relevant to their physicochemical properties, resulting in toxic cell signaling and other neuropathological events, consistent with neurodegenerative diseases and aging [10,11]. In this contest, the lipid composition and physicochemical properties of cell membranes appear to be of the utmost importance, specifically the cholesterol and ganglioside contents that have gained increasing attention in recent years [2,12,13,14]. Gangliosides are glycosphingolipids containing sialic acids (Sias), occurring in human nervous tissue with major species, such as GM1, GD1a, GD1b and GT1b [15]. In several investigations, a significant amount of attention has been focused on specific interactions between different amyloid peptides and gangliosides, particularly between β-amyloid peptides (Aβ) and GM1 [16,17,18]. This is one of the most abundant gangliosides in the brain, representing approximately 20% of their total amount [19,20], and it is a main component of lipid microdomains. GM1 has been shown to bind with and favor the aggregation of Aβ peptides and promote their deposition in senile plaques [21,22,23]. Moreover, a significant alteration in its content was reported in amyloid-positive synaptosomes from AD brains, where lipid microdomains from frontal and temporal cortices were found to contain a higher concentration of GM1, compared to an age-matched control [23]. Of note, Sias seem to be essential for amyloid–GM1 interaction, since depletion of membrane-associated Sia residues protects cells from the toxic effect of exogenous amyloid [2,24]. Sias are neuraminic acid derivatives, which represent a family of large-size negatively charged 9-carbon 2-keto-3-deoxy sugars. They are frequently located in the terminal position in glycan chains of many glycoconjugates, joined to their penultimate sugar acceptor through linkages of different types. Because of these characteristics, a wide variety of pivotal biological functions have been proposed for Sias, such as maintaining membrane stability and modulating several intermolecular and intercellular phenomena in various tissues of animals, including humans, during embryonic and adult life, both under physiological and pathological conditions [25,26,27,28,29,30,31]. Sia linkage types can be α2,3 or α2,6 with galactose and α2,6 with N-acetyl-galactosamine. The type α2-8 linkage is also described to occur between Sia residues and involved in the formation of polysialic acid (PolySia), which is mainly “primed” on an initiating Sia residue α2,3 linked to galactose [32]. Sias with the α2,3/α2,6 linkage are largely diffused among all types of glycoconjugates, such as glycoproteins and glycolipids, particularly gangliosides. Instead, PolySia is mostly linked to the transmembrane glycoprotein Neural Cell Adhesion Molecule (NCAM) [26,28]. PolySia-NCAM is widely distributed in embryonic tissues, poorly expressed in most adult organs and re-expressed or overexpressed in cancer cells, favoring invasiveness and metastasis [26,28,29,30,31,33,34,35,36,37,38,39]. During development, PolySia-NCAM exhibits a high expression, especially in the nervous system, where it promotes dynamic cell interactions involved in neurogenesis, such as cell migration [28,40,41,42,43], axon/dendrite growth and remodeling [40,41,42,43,44] and synaptic reorganization [28,40,41,42,43,44]. Regardless, its expression is evident, although more restricted, in the adult nervous system, where PolySia-NCAM contributes to various forms of neuronal [40,41,42,43,44,45,46] and glial [41,42,43] plasticity, and neural regeneration [41,42,44,47].

Several investigations on Sia expression in post-mortem human brains affected by neurodegenerative diseases, such as AD and PD, or administration of Aβ peptides into the hippocampus of rats reported sialylation dysregulation, particularly in cerebral regions exhibiting tissue plasticity most frequently affected by these pathologies [40,41,46,48,49,50,51,52].

Based on the above background, the present study investigated the sialylation status related to different glycosidic linkages in cell lines, such as human dermal fibroblasts (HDFα) and human neuroblastoma cells both undifferentiated (SH-SY5Y) and differentiation-induced (RA-SH-SY5Y), which vary in their ganglio-tetrosyl ganglioside expression. This research is a groundwork, comparative study that aims at clarifying the relationship between plasma membrane composition, in terms of differently linked Sia contents, and cell susceptibility to amyloid damage. In our opinion, knowledge of this correlation, involving different hierarchical levels, from molecular structure to cell response, might effectively contribute to the understanding of the role of Sias in the development of neurodegenerative diseases.

## 2. Materials and Methods

### 2.1. Aβ-42 Aggregation

Aβ-42 peptide (Bachem, Bubendorf, Switzerland) was dissolved in 100% hexafluoroisopropanol (HFIP) to a final concentration of 1 mM. The solution was aliquoted, HFIP was evaporated and the resulting pellets were stored at −20 °C until needed. These aliquots were stable for at least three months. To generate amyloid aggregates, Aβ-42 peptide was dissolved to a 30 μM concentration in 20 mM sodium phosphate buffer (PBS), pH 7.4, at 25 °C. The samples were sonicated for 15 min and then centrifuged at 14,000× *g* for 15 min at 4 °C. The clear supernatant was collected, and the peptide concentration was checked by evaluating the absorbance of the resulting solution by means of a double-beam Lambda-20 spectrophotometer (Perkin Elmer Life Sciences, Norwalk, CT, USA) (ε280 = 1490 mol^−1^ cm^−1^) and adjusted to a final concentration of 25 μM. Enriched solutions of fibrillar Aβ-42 (Fib) were collected after 72 h of incubation of the supernatant at room temperature (RT).

### 2.2. Cell Cultures

Human neuroblastoma (SH-SY5Y) (CRL-2266, American Type Culture Collection, ATCC, Manassas, VA, USA) cells were cultured in complete medium (50% HAM, 50% DMEM, 10% fetal bovine serum, 3.0 mM glutamine, 100 units/mL penicillin and 100 μg/mL streptomycin), in a humidified 5% CO_2_ incubator at 37 °C. All the materials used for cell culture were purchased by Sigma Aldrich (Milan, Italy). Since neuronal differentiation is accompanied by an increased concentration of the ganglio-tetrosyl gangliosides [53], all-trans-retinoic acid (RA) treatment was exploited on SH-SY5Y cells (RA-SH-SY5Y). For this purpose, cells were cultured for seven days prior to treatment in a differentiation medium containing RA (10 µM) and 3% serum levels [54]. HDFα cells (Thermo Fisher Scientific, Waltham, MA, USA) were cultured in complete medium (DMEM, 10% fetal bovine serum, 3.0 mM glutamine, 100 units/mL penicillin and 100 μg/mL streptomycin at 37 °C in 5% CO_2_). Synaptophysin and NeuN were adopted as markers to evaluate differentiation of neuroblastoma cells.

### 2.3. MTT Assay

Cell viability was evaluated by the MTT assay optimized for the cell line used in the experiments. Briefly, SH-SY5Y, RA-SH-SY5Y and HDFα cells were seeded and grown for 48 h in 96-well plates (6000 cells/well) in their complete medium. Then, cells were treated for 24 h with 2.5 μM of Aβ-42-enriched amyloid fibril solution (Fib). Subsequently, the culture medium was replaced with 100 μL of serum-free DMEM without phenol red, containing 0.5 mg/mL MTT dye, for 1 h at 37 °C. Cell lysis was then achieved by adding 100 µL of lysis solution (20% SDS, 50% N,N-dimethylformamide) to each well, and incubating the samples at 37 °C. Reduction of MTT was evaluated by measuring the absorbance of the blue formazan at 570 nm with a spectrophotometric microplate reader (iMARK microplate reader, Bio-Rad, Hercules, CA, USA). The final absorption values were calculated by averaging each cell preparation in triplicate and subtracting the blank average (100 μL of MTT solution + 100 μL of lysis solution).

### 2.4. Confocal Laser Scanning Microscope Analysis

SH-SY5Y, RA-SH-SY5Y and HDFα cells were cultured with and without Fib solution for different times. GM1 labeling was performed by incubating cells with 10 ng/mL CTX-B Alexa Fluor 488 (Molecular Probes, Eugene, OR, USA) in complete medium for 10 min at RT. Then, cells were fixed in 2% buffered PFA for 10 min, permeabilized by treatment with a 50% acetone/50% ethanol solution for 4 min at RT, washed with PBS and blocked with PBS containing 0.5% bovine serum albumin (BSA) and 0.2% gelatin. To perform PolySia staining, the different cell preparations grown on glass coverslips were fixed, permeabilized, blocked and then incubated overnight at 4 °C with anti-PolySia mouse monoclonal antibody (1:100, LSBio LifeSpan Biosciences, Inc. Seattle, WA, USA). To perform Aβ-42 staining, the cells were incubated with anti-Aβ-42 rabbit polyclonal (1:300, Cell Signaling, Danvers, MA, USA) for 1.5 at RT. The immuno-labeled cells were then washed in PBS and incubated for 1 h at RT with a specific anti-rabbit Alexa Fluor 568-conjugated antibody (1:200, Molecular Probes, Eugene, OR, USA) and anti-mouse Alexa Fluor 488-conjugated antibody (1:200, Molecular Probes, Eugene, OR, USA). Negative controls were carried out by replacing the primary antibody with non-immune serum, whereas the primary antibody was omitted in control experiments for testing the cross-reactivity of the secondary antibody. Coverslips supporting the immuno-labeled cells were washed with PBS, mounted with an antifade mounting medium (Biomeda Gel mount, Electron Microscopy Sciences, Foster City, CA, USA) and observed under a confocal Leica TCS SP5 microscope, equipped with a HeNe/Ar laser source for fluorescence measurements. Observations were performed using a Leica Plan Apo 63X/1.43NA oil immersion objective. Series of optical sections (pixel size 204 nm) were collected through the depth of the specimens at intervals of 0.34 μm, scan frequency 600 Hz, gain values 500–700 (on a 1–1250 scale), line accumulation max 8. Values of laser intensity and gain, as well as number of line accumulations, were settled to better represent the dynamics of the fluorescence intensity of each fluorochrome and maintained constant within each experiment. Images were then superimposed to form a single extended focus image. When needed, differential interference contrast (DIC) fluorescence images were merged. Analysis of the intensity of GM1, PolySia and Aβ-42 fluorescent signals was performed on 3D confocal stacks, using ImageJ software (http://rsbweb.nih.gov/ij (accessed on 7 February 2022)), in 20 regions of interest (ROIs), with surface areas of 100 μm^2^, for each confocal stack (at least 10, i.e., about 600 cells per experimental group). Three-dimensional particle analysis was adopted to isolate Aβ-42 fibrils on the basis of their intensity and size (>0.68 µm^3^). Fluorescence resonance energy transfer (FRET) analysis was performed by adopting the FRET Sensitized Emission Method, as previously reported [55].

### 2.5. Intracellular Calcium

The cytosolic levels of free Ca^2+^ were measured using the fluorescent probe Fluo-3 acetoxymethyl ester (Fluo-3 AM; Molecular Probes-Thermo Fisher Scientific Eugene, OR, USA). Briefly, 7 × 10^4^ HDFα, SH-SY5Y and RA-SH-SY5Y cells were cultured on glass coverslips and incubated at 37 °C for 5 min with 5 μM Fluo-3 AM, prior to 1 h exposure to the Fib sample. At the end of the incubation, cells were fixed in 2% buffered paraformaldehyde (PFA) for 10 min at RT. Cell fluorescence was visualized using a confocal Leica TCS SP8 scanning microscope (Leica Microsystems, Mannheim, Germany) equipped with a HeNe/Ar laser source for fluorescence measurements (excitation at 488 nm). The observations were performed using a Leica Plan 7 Apo X63 oil immersion objective. For further details on acquisition settings, see above. Cells from five independent experiments were analyzed, each involving three coverslip areas (about 20–30 cells/area, 300–450 cells per experimental group). The fluorescence intensity of Fluo-3 AM was analyzed by using ImageJ software (National Institutes of Health, Bethesda, MD, USA) and expressed as arbitrary units.

In parallel, quantification of cytosolic levels of free Ca^2+^ was determined in living cells loaded with Fluo-3 AM by using a fluorescence microplate reader (BioTekSynergy H1, Winooski, VT, USA). HDFα, SH-SY5Y and RA-SH-SY5Y cells were seeded into 96-well culture plates at a density of 10,000 cells/well and allowed to attach for 24 h. After, the cells were incubated at 37 °C for 5 min with 5.0 μM Fluo-3 AM prior to exposure to the Fib sample for 1 h. At the end of the incubation, cell fluorescence was measured at 525 nm (emission wavelength).

### 2.6. Reactive Oxygen Species (ROS) Measurement

The intracellular levels of reactive oxygen species (ROS) were determined using the cell-permeant marker 2′,7′–dichlorofluorescin diacetate, acetyl ester (CM-H2DCFDA; Molecular Probes, Eugene, OR, USA). Fluorescence of CM-H2DCFDA can be elicited only upon removal of the acetate groups by intracellular esterases and subsequent oxidation. For this purpose, HDFα, SH-SY5Y and RA-SH-SY5Y cells were plated at a density of 10,000 cells per well on 96-well plates. After 24 h of cell exposure to the Fib sample, 10 μM CM-H2DCFDA in DMEM without phenol red was added. After 30 min, fluorescence values were measured at 538 nm by Fluoroskan Ascent FL (Thermo-Fisher Scientific, Waltham, MA, USA). Fluorescence was measured in five independent experiments performed in triplicate.

### 2.7. Lectin Histochemistry

Digoxigenin (DIG)-labeled lectins *Maackia amurensis* agglutinin (MAA) and *Sambucus nigra* agglutinin (SNA), contained in DIG Glycan Differentiation Kit (Roche Diagnostic, Mannheim, Germany), were used to identify Sias α2,3 linked to galactose (Gal) and α2,6 linked to Gal or N-acetyl-D-galactosamine (GalNAc), respectively [29,30,56,57,58].

The lectin histochemical technique was performed as previously described [29,30,57]. Briefly, different cell preparations, grown on glass coverslips, were fixed with 0.5% buffered PFA for 10 min at RT, treated with 20% acetic acid for 15 s at 4 °C, to inhibit the endogenous alkaline phosphatase and then incubated with 10% blocking reagent in Tris-buffered Saline (TBS) to reduce the background labeling. Successively, sections were washed in TBS, rinsed in Buffer 1 and then incubated in DIG-labeled lectins diluted in Buffer 1 (1 μL/mL and 5 μL/mL for SNA and MAA, respectively) for 1 h at RT. Afterward, sections were rinsed in TBS, incubated with anti-digoxigenin conjugated with alkaline phosphatase diluted in TBS and then washed in the buffer. The sites containing bound lectin-digoxigenin were labeled incubating slides with Buffer 2, containing nitroblue tetrazolium (NBT)/X-phosphate (Roche Diagnostics, Basel, Switzerland). Control trials for lectin specificity involved substitution of lectin conjugates with the respective unconjugated lectins, or preincubation of lectins with the corresponding hapten sugars (0.1–0.5 M in TBS) [29,30,56,57,58]. The intensity of MAA and SNA lectin reactivity was evaluated by densitometric analysis on digitized images using ImageJ software (http://rsbweb.nih.gov/ij (accessed on 7 February 2022)) in 20 ROIs of 100 μm^2^ for each optical field (at least 10).

### 2.8. Statistical Analysis

All values were tested for normality distribution and were expressed as mean ± standard error of the mean (SE) or standard deviation (SD). A *t*-test or analysis of variance (ANOVA) followed by Tukey’s multiple comparison test, to assess differences among samples, was used. Data analysis was performed using GraphPad Prism 5.0 (GraphPad Software, La Jolla, CA, USA); *p* values < 0.05 were considered statistically significant.

## 3. Results

### 3.1. Evaluation of Different Cell Line Susceptibilities to Aβ-42 Amyloid Toxicity

In order to disclose correlations between the Sia composition of the plasma membrane and cell susceptibility to amyloid damage, a suitable sequence of experimental steps was performed.

**Figure 1:**
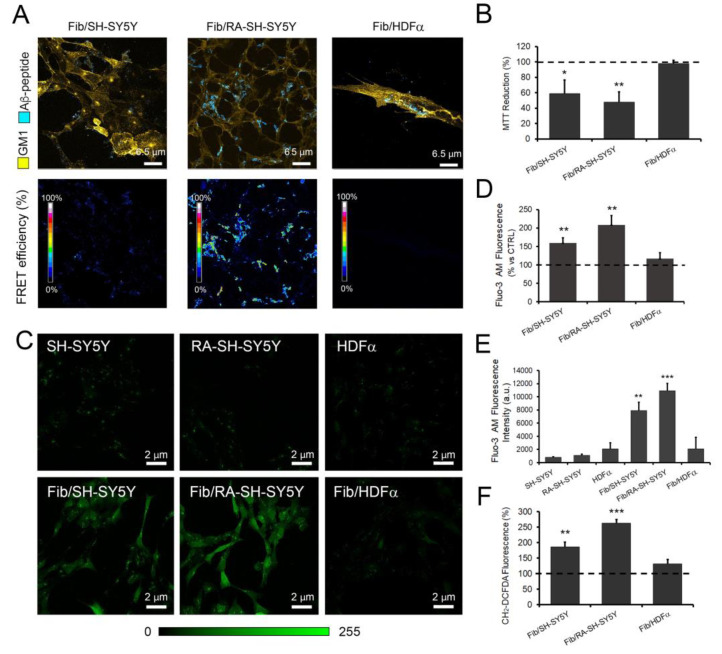
Different cell line susceptibilities to Aβ-42 amyloid toxicity. (**A**) Top panels: Confocal Z-projections of SH-SY5Y, RA-SH-SY5Y and HDFα cells stained to reveal GM1 (yellow) on cell membranes. Cells were treated for 24 h with 2.5 μM (monomer concentration) Aβ-42 Fib solution. Bottom panels: sensitized FRET analysis of GM1 fluorescence staining and fibril immunofluorescence; a 16-color scale was used to represent FRET efficiency. (**B**) Cell viability assessed by the MTT reduction assay on cells treated for 24 h with 2.5 μM Aβ-42 Fib solution: results were expressed as percentage decreases with respect to own controls. Error bars indicate the SD of five independent experiments carried out in triplicate. * *p* < 0.05; ** *p* < 0.01 vs. untreated (control) cells. (**C**) Representative confocal scanning microscopy images of intracellular calcium levels detected in HDFα, SH-SY5Y and RA-SH-SY5Y cells treated for 1 h with 2.5 μM Aβ-42 Fib solution (Fib/HDFα, Fib/SH-SY5Y and Fib/RA-SH-SY5Y) and in control fibroblasts and neuroblastoma cells (HDFα, SH-SY5Y and RA-SH-SY5Y). (**D**) Semiquantitative evaluation of intracellular calcium levels detected by confocal analysis. Variable numbers of cells (60–90) in five independent experiments were analyzed for each condition. (**E**) Semi-quantitative analysis of intracellular free Ca^2+^-derived fluorescence in 96-well plates. Error bars indicate the SD of five independent experiments. ** *p* < 0.01; *** *p* < 0.001 vs. control cells. (**F**) Intracellular ROS production in HDFα, SH-SY5Y and RA-SH-SY5Y cells treated for 24 h with 2.5 μM Aβ-42 Fib solution (Fib/HDFα, Fib/SH-SY5Y and Fib/RA-SH-SY5Y) and in fibroblasts and neuroblastoma cells not treated with amyloid (HDFα, SH-SY5Y and RA-SH-SY5Y). Error bars indicate the SD of five independent experiments carried out in triplicate. ** *p* < 0.01; *** *p* < 0.001 vs. control cells.

Initially, Aβ-42 species’ ability to interact with the plasma membrane ganglioside GM1 was checked. Confocal microscopy analysis revealed an association between Aβ-42 Fib administered for 24 h to cultured cells and the plasma membrane in both undifferentiated and RA-SH-SY5Y lines (Figure 1A), whereas such association was not detected in HDFα fibroblasts (Figure 1A). Moreover, sensitized FRET analysis, assessing aggregate interaction with GM1, revealed higher FRET efficiency in RA-SH-SY5Y than SH-SY5Y cells. The MTT viability assay (Figure 1B) showed that both these cell lines, but not HDFα fibroblasts, were sensitive to the amyloid stressor. In detail, after 24 h of exposure to Aβ-42 Fib, the number of viable neuroblastoma cells was lower than in untreated controls, and RA-SH-SY5Y exhibited lower viability than SH-SY5Y cells. These data stress the view that Aβ-42 binding to plasma membrane GM1 is a hallmark of amyloid-induced cell toxicity [4], possibly due to amyloid internalization, as shown in other neural cell lines [59,60,61].

Successively, intracellular calcium levels were analyzed in neuroblastoma cells and fibroblasts. Both control neuroblastoma and fibroblast lines displayed homogeneous levels of Fluo-3 AM fluorescence (Figure 1C, upper panels), suggesting uniform dye loading in these cells. After 1 h of treatment with Aβ-42 Fib, in HDFα cells, low levels of cytosolic Ca^2+^ comparable to the untreated cells were observed, while an increase in cytoplasmic Ca^2+^ concentrations was detected in the two neuroblastoma cell lines, being significantly higher in RA-SH-SY5Y than in SH-SY5Y cells (Figure 1C–E). This suggested that early interactions of amyloid species with plasma membrane components affected intracellular Ca^2+^ levels, although with obvious differences among cell lines as well as cells of the same line. Indeed, the intensity of Fluo-3 AM fluorescence was remarkably variable among neuroblastoma cells when treated with Aβ-42 Fib, suggesting the heterogeneity of their response to the amyloid stressor (Figure 1C). Moreover, discrete subcellular Ca^2+^ deposits were detected in neuroblasts and fibroblasts regardless of Aβ-42 Fib treatment (Figure 1C, upper panel row), which stresses the constitutive capability of mitochondria and the endoplasmic reticulum (ER) for self-loading with calcium ions.

According to measurements of reactive oxygen species, cytotoxic outcomes of such interactions in both cell lines were confirmed by increased ROS levels found after 24 h of treatment. Of note, these values were higher in RA-SH-SY5Y than in SH-SY5Y cells (Figure 1D), thus confirming the higher susceptibility to the amyloid insult of RA-treated cells in comparison with their untreated counterparts. By contrast, in HDFα fibroblasts, no significant ROS increase was observed (Figure 1F).

### 3.2. MAA and SNA Reactivity

**Figure 2:**
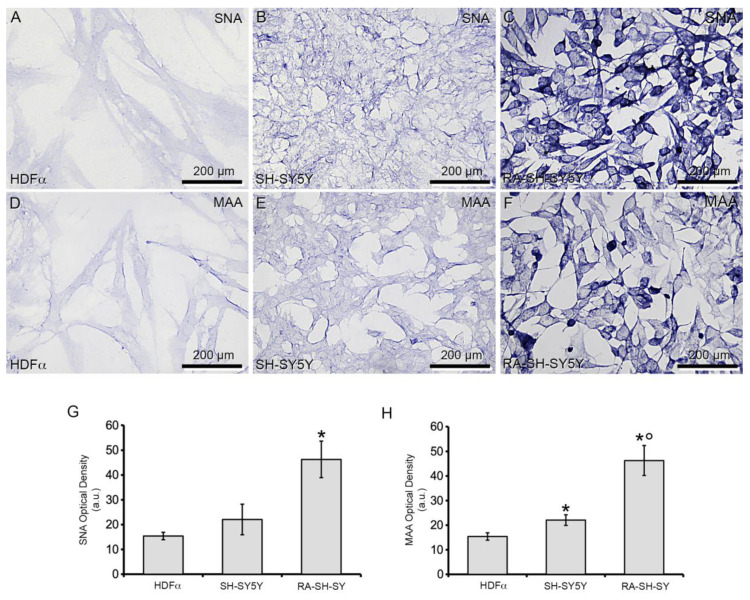
SNA and MAA lectin reactivity. (**A**–**C**) SNA and (**D**–**F**) MAA reactivity in HDFα, SH-SY5Y and RA-SH-SY5Y, to reveal Sias α2,6 linked to Gal/GalNAc and α2,3 linked to Gal, respectively. (**G**,**H**) Quantitative analysis of SNA (F_(2,6)_ = 109.01, * *p* < 0.05 vs. HDFα) and MAA (F_(2,6)_ = 90.6, * *p* < 0.05 vs. HDFα, ° *p* < 0.01 vs. SH-SY5Y) reactivity. Data are reported as averages ± SE of three independent experiments.

MAA and SNA DIG-labeled lectins were then employed in histochemical trials to evaluate, respectively, the expression of α2,3 Gal-linked Sias, characteristic of most gangliosides, especially GM1, and α2,6 Gal/GalNAc-linked Sias, found in other gangliosides [62]. In all cell lines investigated, MAA and SNA reactivity was observed along cell profiles, indicating plasma membrane localization. Significantly, RA-SH-SY5Y cells showed stronger responses both with SNA and MAA lectins with respect to SH-SY5Y and HDFα cells. Comparing these less reactive lines, SH-SY5Y cells’ reactivity was stronger than that of HDFα, but significantly only with MAA lectin (Figure 2). In addition, a remarkable variability of SNA and MAA reactivity was observed among RA-SH-SY5Y cells, which exhibited intense staining both in their somata and cytoplasmic processes. In tests of the control for lectin specificity, both sections incubated with lectins plus their corresponding hapten sugars, and sections with unconjugated lectins were unstained (data not shown).

### 3.3. Confocal Microscopy of PolySia and Aβ-42 Amyloid Immunoreactivity

**Figure 3:**
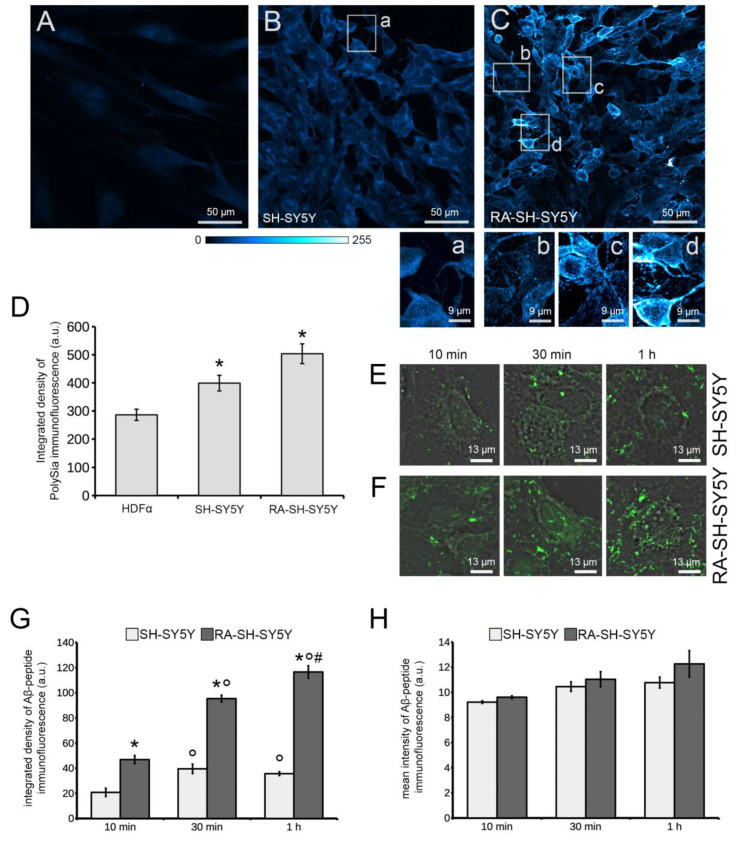
PolySia-specific mAb and Aβ-42 amyloid polyAb immunoreactivity. (**A**–**C**) PolySia immunoreactivity (cyan) in HDFα, SH-SY5Y and RA-SH-SY5Y cells. Insets (**a**–**d**) show details of the corresponding areas selected in ((**B**)/(**a**),(**C**)/(**b**–**d**)), showing PolySia immunofluorescence in cytoplasm processes. (**D**) Quantitative analysis of PolySia immunoreactivity; *n* = 3, mean values of integrated density of immunofluorescence ± SE are shown, * *p* < 0.05 vs. HDFα. (**E**,**F**) Immunoreactivity of Aβ-42 Fib seeded on SH-SY5Y (**E**) and RA-SH-SY5Y (**F**) for 10 min, 30 min and 1 h; merged Aβ-42 immunofluorescence (green) and DIC images are shown. (**G**,**H**) Quantitative analyses of Aβ-42 immunofluorescence; values of integrated density (**G**) and mean intensity (**H**) of immunofluorescence are shown; * *p* < 0.01 vs. SH-SY5Y, two-way ANOVA F_(1,12)_ = 415.5; ° *p* < 0.01 vs. 10 min, two-way ANOVA F_(2,12)_ = 90.1, ^#^ *p* < 0.01 vs. 30 min, two-way ANOVA F_(2,12)_ = 90.1. Data are reported as the average ± SE of three independent experiments.

The expression of PolySia was revealed by immunofluorescence staining and analyzed by confocal microscopy. As expected from the above findings, the immunoreactivity of PolySia was significantly lower in HDFα cells than in the neuronal lines, where RA-SH-SY5Y showed stronger fluorescence than SH-SY5Y cells (Figure 3A,D). In the three cell lines, the immunofluorescence of PolySia was localized in peri-nuclear intracellular regions and along cell profiles (Figure 3A–C).

Interestingly, levels of the fluorescence intensity showed remarkable variability among RA-SH-SY5Y cells, as observed in evaluating SNA and MAA reactivity. Moreover, PolySia in the plasma membrane of RA-SH-SY5Y cells showed a less uniform distribution than in the other two cell lines, being concentrated in small areas of a high fluorescence density, and along edges of somata and outlines of newly formed cytoplasmic processes (Figure 3 insets b–d). In control trials, sections were unreactive both by replacing the primary antibody with non-immune serum, and by omitting the primary antibody (data not shown). The above data suggest that RA treatment affects the PolySia distribution in neuroblastoma cells. In a further step, early differential deposition of amyloid fibrillar aggregates was, therefore, evaluated on specimens at succeeding stages of cytodifferentiation, namely, undifferentiated and RA-differentiated neuroblastoma cells, whereas HDFα cells were no longer investigated in this study.

Aβ-42 Fib seeded on SH-SY5Y and RA-SH-SY5Y cells after 10 min, 30 min and 1 h exposures were immunostained and analyzed by confocal microscopy (Figure 3E–H). In both cell groups, higher deposition of Aβ-42 was more evident after 30 min than after 10 min of treatment (Figure 3E,F), as confirmed by quantitative analysis of the integrated density of the immunofluorescent signal (Figure 3G), expressing the total amount of fluorescence measured within the analyzed field of view. Furthermore, the amount of deposits was higher in RA-SH-SY5Y than in SH-SY5Y cells (Figure 3E–G). Moreover, a significant increase in amyloid deposition was evident in the former, but not in the latter, cell line, after 1 h of treatment when compared with 30 min (Figure 3E–G). Quantitative analysis of the mean intensity of Aβ-42 immunofluorescence did not show any significant difference either among different time points or differentiation stages (Figure 3H), suggesting the structural homogeneity of Aβ-42 deposits.

### 3.4. Differential Binding of GM1 with Aβ-42 Amyloid in SH-SY5Y and RA-SH-SY5Y Cells

**Figure 4:**
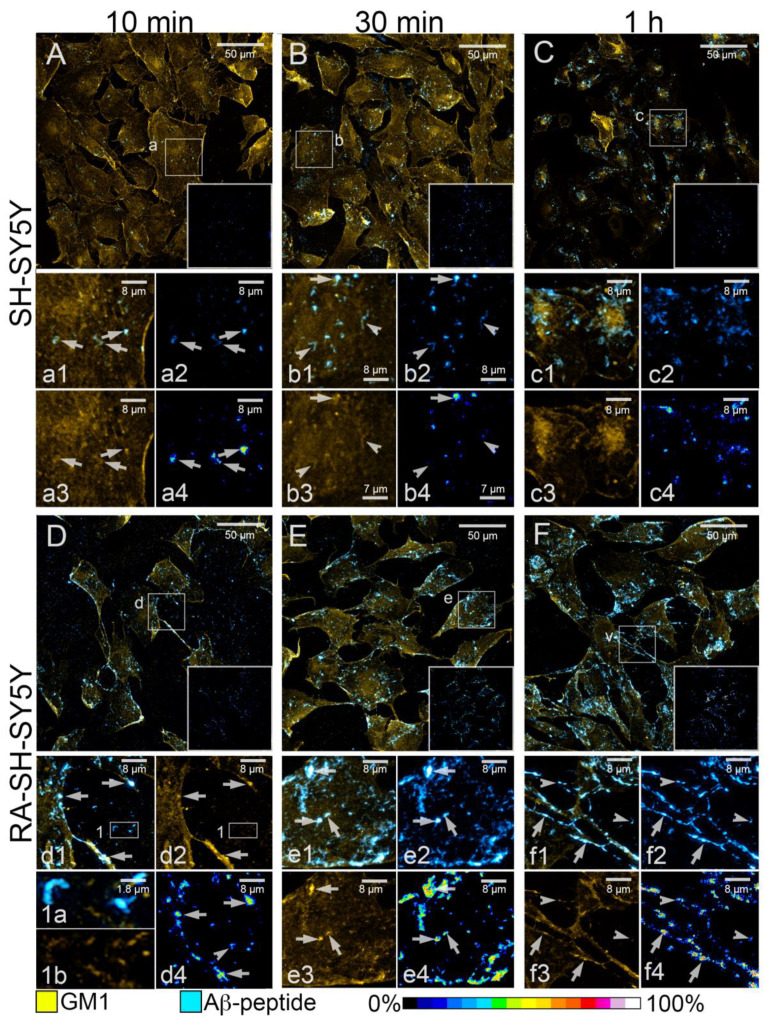
GM1 and Aβ-42 amyloid species in SH-SY5Y and RA-SH-SY5Y cells. (**A**–**F**) Representative images of SH-SY5Y (**A**–**C**) and RA-SH-SY5Y (**D**–**F**) cells stained to reveal GM1 (Yellow) after 10 min (**A**,**D**), 30 min (**B**,**E**) and 1 h (**C**–**F**) of incubation with Aβ-Fib (Cyan); square areas in lower right corner insets show efficiency of FRET interactions (corresponding full-size images are shown in Supplementary Figure S1). Insets (**a**–**e**,**v**) show details of the areas selected in images (**A**–**F**): images of merged channels (**a1**–**f1**), Aβ-42 immunofluorescence (**a2**–**f2**), GM1 staining (**a3**–**c3**,**e3**,**f3**) and FRET efficiency (**a4**–**f4**) are shown. Arrows in (**a**–**f**) indicate sites of high FRET efficiency between amyloid species and gangliosides. Arrowheads in (**a**–**c**) indicate sites of low FRET efficiency. Arrowheads in (**d**–**f**) indicate sites of amyloid binding to cell fragments on glass coverslips. Inset 1 shows details of the area selected in (**d)**; merged fluorescent signals (**1a**) and GM1 staining alone (**1b**) are shown.

**Figure 5:**
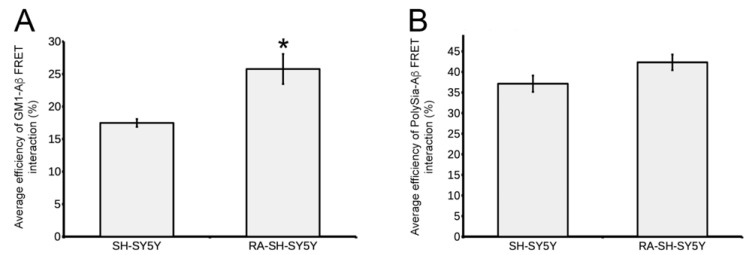
Comparative analysis of FRET interactions. Efficiency of FRET interactions between the fluorochrome Alexa Fluor 488 on GM1 (**A**) and PolySia (**B**) with Alexa Fluor 568 on Aβ-42 species adhering to the plasma membrane of SH-SY5Y and RA-SH-SY5Y cells after 1 h of seeding. Values are expressed as average ± SE; *n* = 3. T-test * *p* < 0.05 vs. SH-SY5Y.

The data above show that differences in sialylation status may underlay the different affinities of RA-treated neuroblastoma cells to Aβ-42 amyloid species with respect to their untreated counterparts and significantly affect early binding reactions of such aggregates on plasma membranes. Therefore, following the above timing sequence, the binding of GM1, exposing the α2,3 Gal-linked Sia on its extracellular moieties, with Aβ-42 was evaluated in SH-SY5Y (Figure 4A–C) and RA-SH-SY5Y (Figure 4D–F) cell cultures after 10 min, 30 min and 1 h exposures. In SH-SY5Y samples after 10 and 30 min treatments, the fluorescence of GM1 was diffused over the whole cell surface, disclosing morphological traits of undifferentiated neuroblastoma cells: round-shaped cell bodies with few, short and thin cytoplasmic processes (Figure 4A,B). Discrete sites of GM1 reactivity were detectable at cell edges, and in small dots distributed on the exposed surfaces (Figure 4 insets a3,b3). Aβ-42 aggregates appeared either disorganized or shaped as short filaments and were frequently associated with the assemblies of GM1 (Figure 4 insets a1–a3,b1–b3). Such an association was confirmed by colocalization analyses, indicating high fractions of Aβ-42 immunofluorescence overlapping the fluorescent staining of GM1 in all the analyzed cell groups (Supplementary Table S1). These data stress the role of this ganglioside in mediating the adhesion and binding of Aβ-42 amyloid aggregates to the plasma membrane. However, despite a consistent localization of GM1 at cell edges, Aβ-42 species were found to mainly bind with its small assemblies on cell surfaces at the 10 min time point (Figure 4A and insets a1,a3). A similar pattern of Aβ-42 distribution was observed at the 30 min time point (Figure 4B). These data indicate different local affinities between Aβ-42 and GM1 depending on its localization and suggest the involvement of a further mediator of Aβ-42 binding to the plasma membrane. This latter hypothesis was supported by analysis of FRET interactions between the fluorochromes associated with GM1 and Aβ-42 on cell surfaces. On the one hand, diffuse FRET signals were observed in SH-SY5Y cells (inner insets in Figure 4A,B and insets a4,b4; Supplementary Figure S1A,B), with the highest efficiency of about 50%. On the other hand, several Aβ-42 aggregates were found on these cells, showing an almost null FRET interaction with GM1 (Figure 4 insets b2,b4). After 1 h of exposure to amyloid species, GM1 staining was concentrated in the central areas of cell surfaces (Figure 4 insets c1,c3) and was encircled and crossed by amorphous Aβ-42^+^ aggregates (Figure 4 insets c1,c2), emphasizing the ability of amyloid fibrils to affect the plasma membrane localization of GM1. A high variability of FRET interactions was also found in SH-SY5Y cells at this time of seeding (inner inset in Figure 4C and inset c4; Supplementary Figure S1C). The average FRET efficiency was about 18% (Figure 5A), and values did not show any statistical difference, when compared in different time points of treatment (data not shown).

RA-SH-SY5Y specimens were characterized by a lower cellular density when compared with their undifferentiated counterparts. Their bodies were generally smaller, and GM1^+^ processes of varying thickness (from ≈0.4 to ≈1.5 µm) extended from the somata, frequently contacting other cells (Figure 4D–F and insets d1,d2,f1,f3). Similar to undifferentiated SH-SY5Y cells, high densities of GM1 fluorescence were found at cellular edges (Figure 4D–F) and in dots on cell surfaces (Figure 4 inset e3). Of note, RA-SH-SY5Y cells showed exclusive GM1 staining on their processes (Figure 4 insets d2,f3). Moreover, numerous cell fragments showing GM1 staining were observed adhering to glass coverslips (Figure 4 insets 1a,1b). Immunofluorescent Aβ-42 species on RA-SH-SY5Y cells appeared more regularly shaped (filamentous), and longer than in undifferentiated SH-SY5Y cells (Figure 4D–F and inserts d1–f1,e2,f2). Interestingly, GM1 in RA-treated cells, when compared with their untreated counterparts, displayed regionally different specific affinities to amyloid fibrils: along with Aβ-42 deposits on their surfaces, Aβ-42 immunoreactivity was predominantly detected at cell edges at all the observed time points (Figure 4D–F and insets d1,e1). Moreover, consistent localizations of Aβ-42 aggregates were observed on cytoplasmic projections (Figure 4D–F and insets d1,f1) as well as cell fragments (Figure 4 insets 1a,1b). The fraction of the GM1 fluorescent signal overlapping the immunofluorescence of Aβ-42 species was significantly higher in RA-treated neuroblastoma cells than in their untreated counterparts (Supplementary Table S1). The efficiency of FRET interactions in RA-SH-SY5Y cells (about 26%) was significantly higher than in SH-SY5Y cells (about 18%; Figure 5A). In detail, the highest FRET efficiency was found at cell edges and on cytoplasmic processes (inner insets in Figure 4 and insets d4–f4; Supplementary Figure S2D–F), whereas remarkably variable levels were found on cell somata (Figure 4 insets e2,e4), thus confirming the effect exerted by RA treatment on the regionally specific affinity of GM1/Aβ-42 binding.

### 3.5. PolySia Affects Aβ-42/Plasma Membrane Binding in SH-SY5Y and RA-SH-SY5Y Cells

**Figure 6:**
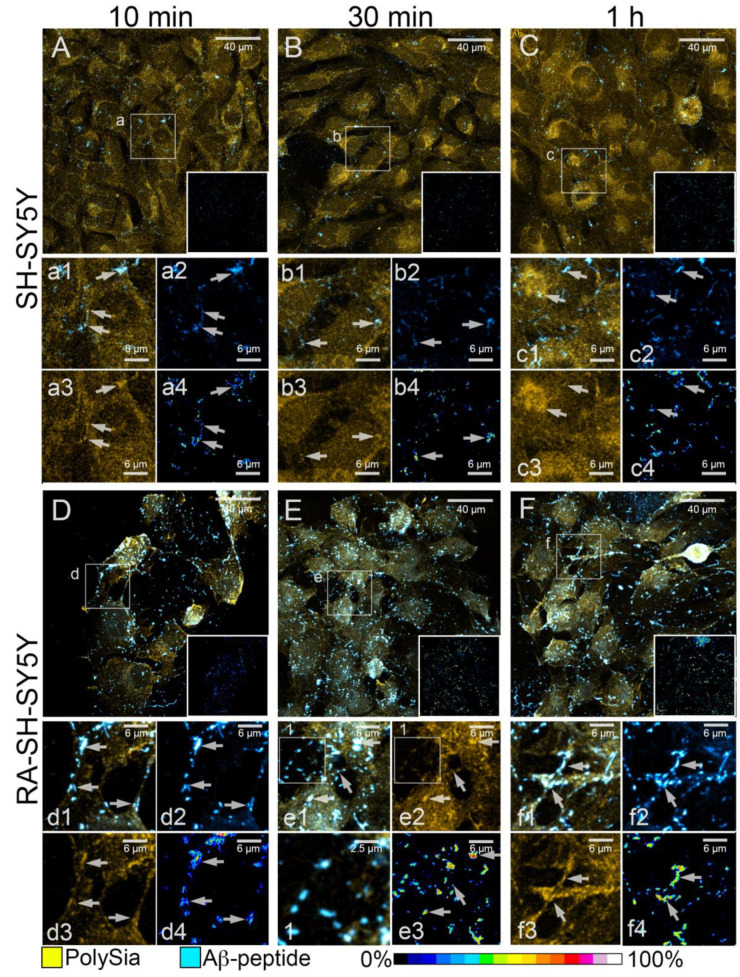
PolySia and Aβ-42 amyloid in SH-SY5Y and RA-SH-SY5Y cells. (**A**–**F**) Representative images of SH-SY5Y (**A**–**C**) and RA-SH-SY5Y (**D**–**F**) cells stained to reveal PolySia (Yellow) after 10 min (**A**,**D**), 30 min (**B**,**E**) and 1 h (**C**–**F**) of incubation with Aβ species (Cyan); square areas in lower right corners show the efficiency of FRET interactions (corresponding full-size images are shown in Supplementary Figure S2). Insets (**a**–**f**) show details of the areas selected in images (**A**–**F**): images of merged channels (**a1**–**f1**), Aβ-42 immunofluorescence (**a2**–**d2**,**f2**), PolySia staining (**a3**–**d3**,**e2**,**f3**) and FRET efficiency (**a4**–**d4**,**e3**,**f4**) are shown. Arrows in (**a**–**c**) indicate sites of high FRET efficiency between amyloid species and PolySia on cell bodies. Arrows in (**d**–**f**) indicate sites of binding between Aβ-42 and PolySia on cytoplasmic projections. Inset 1 shows details of the area shown in (**e1**,**e2**); merged fluorescent signals and PolySia staining alone are shown.

Confocal analyses were also performed on neuroblastoma cells immunostained to reveal both Aβ-42 aggregates and cell surface PolySia (Figure 6). Deposition of Aβ-42 species did not affect PolySia localization at the different time points in SH-SY5Y (Figure 6A–C, Supplementary Figure S3A–C) as well as in RA-SH-SY5Y cells (Figure 6D–F, Supplementary Figure S2D–F). FRET interactions were found to occur between PolySia and almost all amyloid deposits on plasma membranes of both cell lines, and their average efficiency was about 40% (Figure 5B, inner insets in Figure 6A–F and Supplementary Figure S3A–F). These data suggest that binding of PolySia and Aβ-42 was not characterized by regionally specific affinity. Of note, high densities of PolySia immunofluorescence in RA-SH-SY5Y cells were found at the adhesion sites of amyloid species, at cell edges, on cytoplasmic processes and in cell fragments on glass coverslips (Figure 6D–F and insets d–f). Accordingly, a high efficiency of FRET interactions between the fluorochromes linked to PolySia and Aβ-42 species was found at these sites (Figure 6 insets d4, e3, f4). On account of these data, it can be proposed that different localizations of PolySia in various cell lines may play a role in the regionally specific affinity of Aβ-42/plasma membrane binding.

## 4. Discussion

This study reports, for the first time, the relationships between differential Sia contents of the plasma membrane and cell susceptibility to amyloid damage. For this purpose, the sialylation status of the plasma membrane was evaluated in different cell models: HDFα, undifferentiated SH-SY5Y and RA-SH-SY5Y, which showed different expressions of gangliosides. Overall, our findings demonstrate that expressions of Sias, both α2,3 and α2,6 linked, and polySia are positively correlated with cell susceptibility to the amyloid insult. Importantly, the data here also stress the relevance of both the expression and localization of PolySia in the early phase of interaction between Aβ-42 amyloid species and the plasma membrane. Preliminary analyses showed that the intrinsic toxicity of Aβ-42 amyloid aggregates is associated with their binding to membrane GM1. This toxicity was estimated by a number of parameters such as intracellular free calcium levels, ROS production and cell viability, which are remarkably altered in RA-SH-SY5Y cells.

Indeed, GM1 is the most investigated among major ganglioside species, due to its property of binding to amyloid and favoring both the deposition and aggregation of Aβ peptides in brain aging as well as neurodegenerative diseases [2,62,63,64,65]. However, other ganglioside species have been reported to be involved in these processes [62]. Of note, GM1 and most of these gangliosides are characterized only by α2,3 Gal-linked Sia, whereas a minority of other species also exhibit α2,6 Gal/GalNAc-linked Sia (62). Interestingly, lectin histochemistry, performed in this study, revealed that the expression of Sia, both α2,3 and α2,6 linked to galactose/galactosamine, is higher in neuroblastoma cell lines, noticeably in RA-SH-SY5Y compared to HDFα cells. In addition, RA-SH-SY5Y cells display significant variability of Sia expression, probably due to the different levels of the differentiation process that is induced in them. Indeed, Sias linked to gangliosides are essential for cell interactions with the microenvironment and seem to be involved in fundamental neurobiological processes performed by these glycolipids, such as axonal and dendritic growth, synapse formation and synaptic transmission [48,65,66,67,68,69,70]. Among RA-SH-SY5Y cells, which show neuronal-like plasticity, higher levels of Sias may therefore be expressed at more advanced differentiation stages.

It should be recalled that Sias reported in the human central nervous system (CNS) occur not only in gangliosides (65%), but also in glycoproteins (32%), and in the free form (3%) [48].

In view of such different percentages, this study also investigated the possible role of the polymeric form, PolySia, which is mostly linked to NCAM, a glycoprotein widely expressed in the nervous system [29,40,41,42,43,45]. Polysialylation of NCAM, modulating its adhesive property [40], supports its role in neural plasticity. Indeed, this molecular process is involved in morphological–functional changes, such as neurogenesis, cell migration, axon/dendrite growth and remodeling and synaptic reorganization, its role spanning from development to adult life [40,41,49,50,65]. Furthermore, PolySia is also strongly expressed in cancer cells favoring invasiveness and metastasis [26,31]. On account of their neuronal and oncological traits, neuroblastoma lines represent cell models provided with high plasticity and are suitable for investigating the variability of PolySia expression [35,70]. As expected from their specific differentiation perspectives, our results show higher levels of PolySia in both neuroblastoma cell lines, especially in RA-SH-SY5Y compared to HDFα cells. The higher levels of PolySia detected in peri-nuclear regions of RA-SH-SY5Y cells in comparison with SH-SY5Y suggest, in the former line, a stronger activation of biosynthesis processes, reasonably involved in cell plasticity. Moreover, PolySia expression, as observed in α2,3 and α2,6 linked Sias, shows remarkable variability among RA-SH-SY5Y cells, confirming that higher levels of sialylation play crucial roles in differential expression patterns of neuronal-like plasticity. Of note, it has been suggested that neuronal plasticity variation and PolySia overexpression in the hippocampus are integral components of the pathologic cascade in AD [49,50]. It may be a molecular, adaptive response from neurons that compensates damages caused by the disease, preserving their integrity and restoring their functionality through dynamic processes of neurogenesis, as well as remodeling, reorganization and/or regeneration of neurites [41,48,49,50,51]. To recall, discrete areas of PolySia at a high density were localized in RA-SH-SY5Y cells at soma edges and cytoplasmic projections as well. Accordingly, the recruitment of such molecules on cytoplasmic outgrowths of differentiating neuroblastoma cells involved in re-shaping is suggestive of the involvement of PolySia in the formation of neuronal processes.

Overall, the above data indicate that sialylation status in the three cell models could be relevant to their different binding propensities with amyloid fibrils and related susceptibility to the amyloid insult. It is known that the plasma membrane may play a significant role in triggering nucleation, pathological aggregation and accumulation of amyloid fibrils, and therefore in cell susceptibility to amyloid injury. Moreover, in neurodegenerative diseases, this interaction with the cell membrane also plays a crucial role for amyloid peptides spread from one cell to another and promotes nucleation, recruiting proteins in the misfolded form to form growing aggregates that may exert further cytotoxic effects in nearby cells [71,72,73,74]. Our time-lapse analysis of Aβ-42 seeding in neuroblastoma cell lines showed that differences in binding affinity become evident even after 10 min exposure to the amyloid stressor. In these early interactions, the GM1 ganglioside displays regionally specific, differential affinity towards Aβ-42, which is higher in RA-SH-SY5Y cells, involving cell edges and cytoplasm processes. Reasonably, GM1 in different cell regions may be involved in locally specific roles, which means that its binding with amyloid fibrils may be affected by an association with functional molecules differentially expressed in each area. Starting from such an inference, the involvement of PolySia in Aβ-42/plasma membrane interactions was specifically investigated. The binding affinity between the molecules engaged in such interactions is high and shows no regional specificity in the two neuroblastoma cell lines. Moreover, the consistent localization of Polysia at cell edges and on cytoplasmic processes observed in the RA-SH-SY5Y cells is positively correlated with the seeding of the amyloid fibrils in these areas. It is noteworthy that the increase in amyloid deposition at the early time point does not correlate with the PolySia level rise in both cell lines, thus confirming our observations to address plasma membrane/Aβ-42 binding affinity. In view of these data, it can be assumed that the concomitance of PolySia-NCAM, GM1 and other possible gangliosides and glycoproteins may be relevant to the early binding of Aβ-42 with the plasma membrane and play a crucial role in triggering and advancing AD neurodegeneration.

In conclusion, our findings demonstrate that cells highly susceptible to amyloid Aβ-42 cytotoxicity, such as the RA-SH-SY5Y neuroblastoma line, which expresses a prevailing plastic neuron-like phenotype, also display high monoSia and PolySia contents. In addition, PolySia shows strong affinity with Aβ-42 fibrils in early phases of their interaction, which favors amyloid binding to GM1, containing α2,3 Gal-linked Sia, and leads to a drop in cell viability. Possibly, other gangliosides and glycoproteins, characterized by both α2,3 Gal-linked Sia and α2,6 Gal/GalNAc-linked Sia, may interact with amyloid and increase its recruitment. We hypothesize that the high expression of Sias in neurons occurring in brain plastic areas could significantly favor their binding to amyloid fibrils. Successively, the amyloid aggregates, along with other destabilizing factors (ROS species, debris from damaged mitochondria, etc.) originating from nerve cells, could lead to a change in their metabolism. Up-regulation of Sia biosynthesis in nerve cells may be involved in this multifactorial context, promoting a compensative/protective increase in PolySia. This, in turn, could favor amyloid binding to Sia-linked gangliosides, such as GM1, thus exacerbating cell dysmetabolism and promoting amyloid aggregation. In this view, investigations of Sia and PolySia contents in different brain regions may disclose the role of such molecules in diverse neurodegenerative diseases and significantly contribute to the development of novel therapeutic approaches.

## Data Availability

Not applicable.

## Supplementary Materials

The following are available online at https://www.mdpi.com/article/10.3390/cells11040601/s1, Figure S1: Immunofluorescence analysis of neuronal differentiation markers; Figure S2: FRET interactions occurring between GM1 and Aβ-42 amyloid. Figure S3: FRET interactions occurring between PolySia and Aβ-42 amyloid. Table S1: Quantitative analysis of colocalization between Aβ 42 immunofluorescence and GM1 fluorescent staining.
