# Supplementary material for: Correlation between Sialylation Status and Cell Susceptibility to Amyloid Toxicity
Source: Cells. 2022 Feb 9;11(4):601. doi: 10.3390/cells11040601 (PMC8870280; doi:10.3390/cells11040601)
Supplement: Supplementary file 1 [file cells-11-00601-s001.zip › cells-1521909-supplementary.pdf]

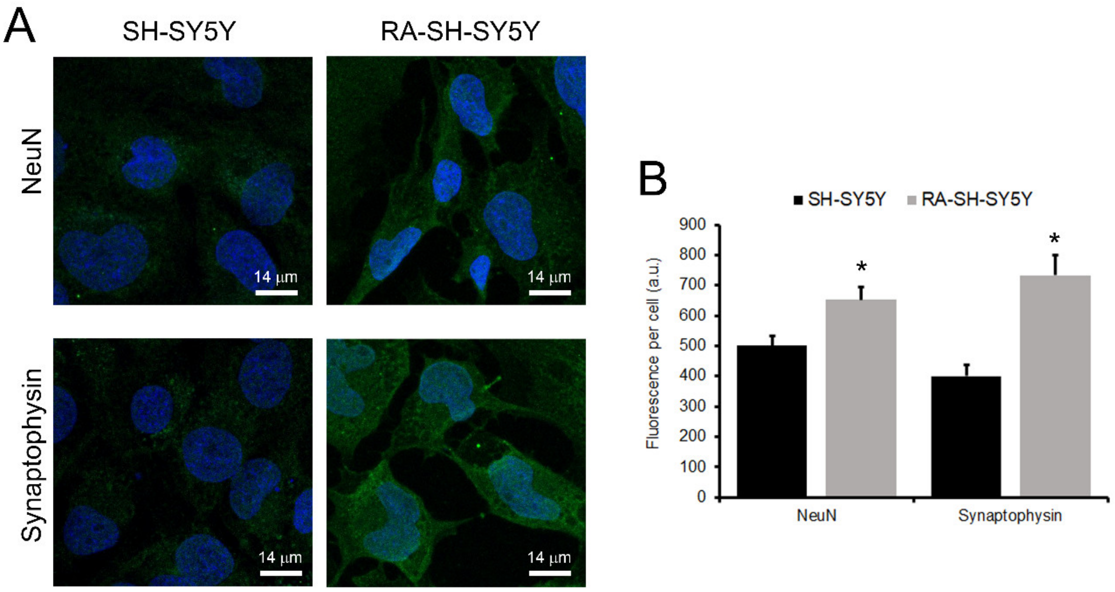

Figure S1. Immunofluorescence analysis of neuronal differentiation markers. Immunofluorescence analysis (A) and quantification of fluorescence signals (B) in SH-SY5Y and RA-SH-SY5Y neuroblastoma cells stained with Hoechst 33258 and with anti-neuronal maturation protein, NeuN, and anti-Synaptophysin (Syn). p *< 0.05 vs SH-SY5Y undifferentiated cells.

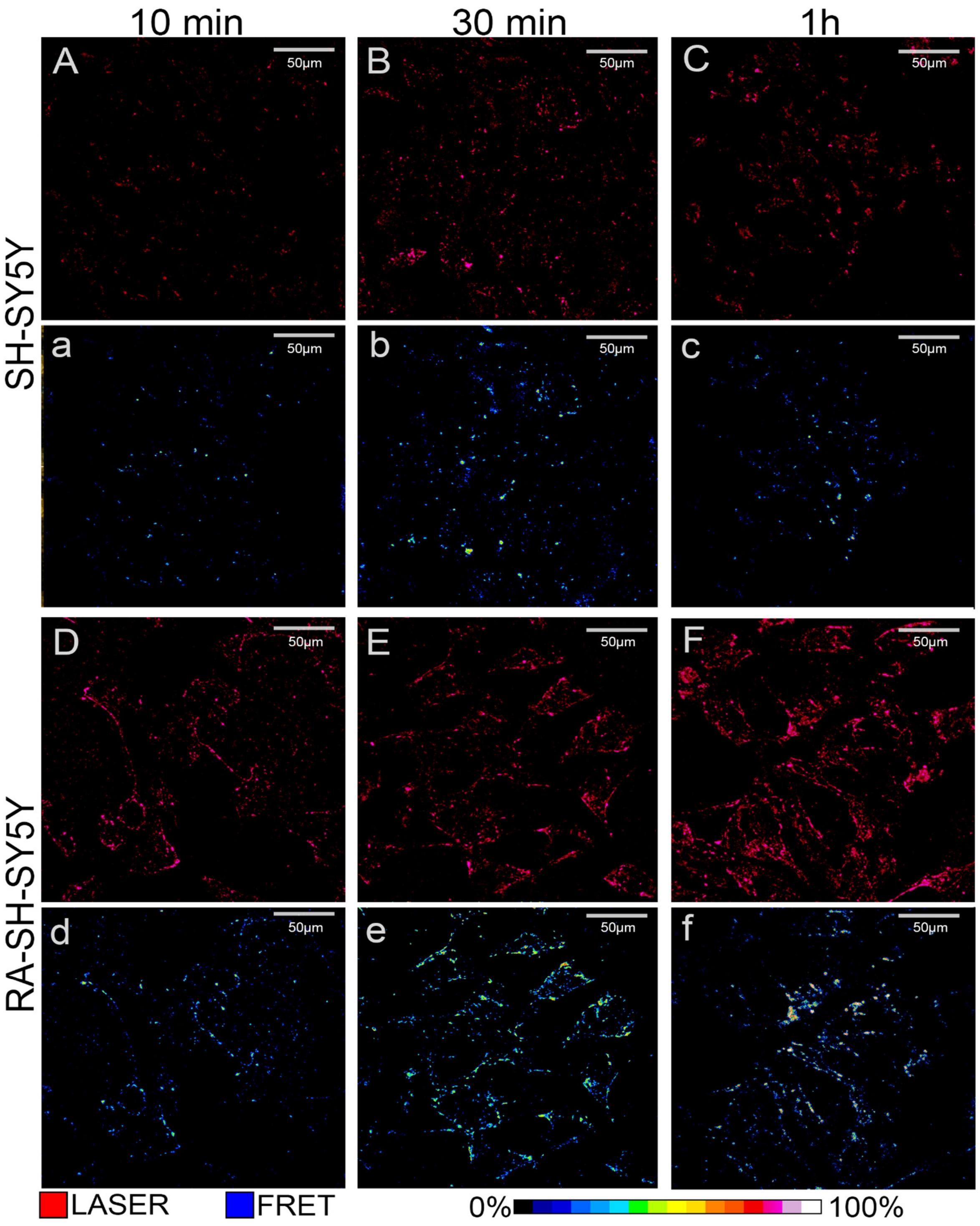

Figure S2. FRET interactions occurring between GM1 and Aβ-42 amyloid. (**A**–**F**) Immunofluorescence of Alexa Fluor 568 on immuno-labeled Aβ-42 amyloid deposits excited by 543 laser line (red) and FRET donor Alexa Fluor 488 on GM1 (blue) on SH-SY5Y (**A**–**C**) and RA-SH-SY5Y (**D**–**F**) cells from Figure 4; insets (**a**–**f**) show the corresponding FRET efficiency.

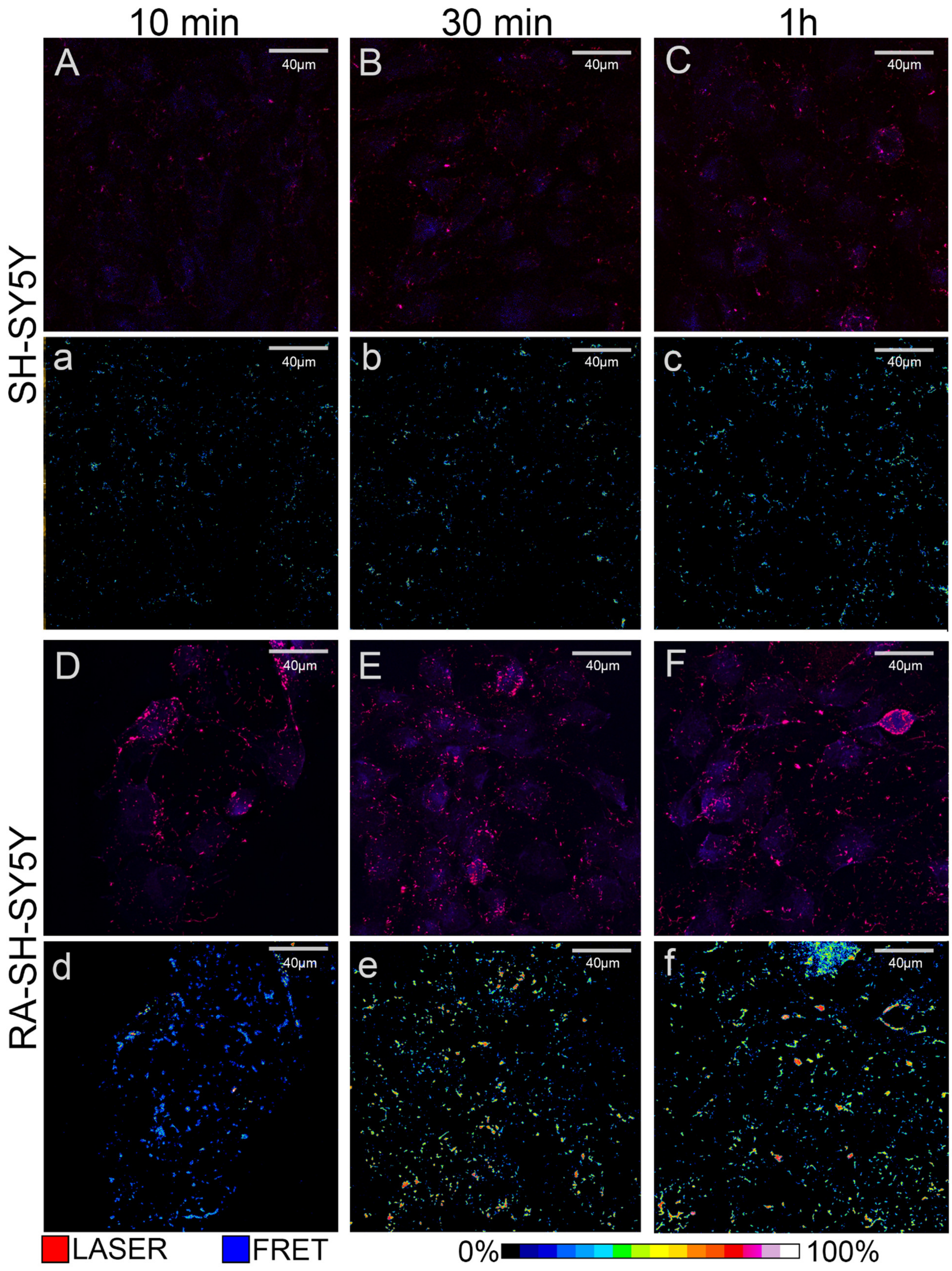

Figure S3. FRET interactions occurring between PolySia and Aβ-42 amyloid. (**A**–**F**) Immunofluorescence of Alexa Fluor 568 on immuno-labeled Aβ-42 amyloid deposits excited by 543 laser line (red) and FRET donor Alexa Fluor 488 on PolySia (blue) on SH-SY5Y (**A**–**C**) and RA-SH-SY5Y (**D**–**F**) cells from Figure 5; insets (**a**–**f**) show the corresponding FRET efficiency.

Table S1. Quantitative analysis of colocalization between Aβ 42 immunofluorescence and GM1 fluorescent staining. Mander's coefficients M1 (fraction of GM1 signal overlapping Aβ 42 signal) and M2 (fraction of Aβ 42 signal overlapping GM1 signal) were estimated. Two-way ANOVA followed by Bonferroni post hoc test was adopted to perform statistical analyses, the 2 independent variables being the RA treatment (n = 18) and the different times of exposure to amyloid fibrils (n = 3). Assuming M1 as the dependent variable: * $p < 0.01$ vs SH-SY5Y cells; ° $p < 0.05$ vs SH-SY5Y 10 m, § $p < 0.05$ vs SH-SY5Y 30 m; $F_{(5,12)} = 31$. Assuming M2 as the dependent variable: $F_{(5,12)} = 0.55$.

	M1	M2
SH-SY5Y	0.35 ± 0.02	0.55 ± 0.03
RA-SH-SY5Y	0.35 ± 0.02 *	0.63 ± 0.05
SH-SY5Y 10 min	0.11 ± 0.003	0.58 ± 0.01
RA-SH-SY5Y 10 min	0.32 ± 0.003 °§	0.55 ± 0.02
SH-SY5Y 30 min	0.1 ± 0.003	0.51 ± 0.03
RA-SH-SY5Y 30 min	0.31 ± 0.007 °§	0.67 ± 0.08
SH-SY5Y 1 h	0.23 + 0.01 °§	0.57 ± 0.05
RA-SH-SY5Y 1 h	0.38 ± 0.02 °§	0.66 ± 0.08
